# Severe toxicity from checkpoint protein inhibitors: What intensive care physicians need to know?

**DOI:** 10.1186/s13613-019-0487-x

**Published:** 2019-02-01

**Authors:** Virginie Lemiale, Anne-Pascale Meert, François Vincent, Michael Darmon, Philippe R. Bauer, Andry Van de Louw, Elie Azoulay, Groupe de Recherche en Reanimation Respiratoire du patient d’Onco-Hématologie (Grrr-OH)

**Affiliations:** 1 Medical Intensive Care Unit, APHP, Hôpital Saint-Louis, Paris, France; 2 Soins Intensifs et urgences oncologiques, Institut Jules Bordet (ULB), Brussels, Belgium; 3 Medical Surgical ICU, GHIC Le Raincy-Montfermeil, 93370 Montfermeil, France; 4 ECSTRA, Saint Louis SBIM, APHP, Paris, France; 5 Pulmonary and Critical Care, Mayo Clinic, Rochester, MN USA; 6 Division of Pulmonary and Critical Care Medicine, Penn State University College of Medicine, Hershey, PA USA

**Keywords:** Immunotherapy, Cancer, ICU, Adverse events

## Abstract

Checkpoint protein inhibitor antibodies (CPI), including cytotoxic T-lymphocyte-associated antigen 4 inhibitors (ipilimumab, tremelimumab) and the programmed cell death protein 1 pathway/programmed cell death protein 1 ligand inhibitors (pembrolizumab, nivolumab, durvalumab, atezolizumab), have entered routine practice for the treatment of many cancers. They improve the outcome for many cancers, and more patients will be treated with CPI in the future. Although CPI can lead to adverse events (AE) less frequently than for chemotherapy, their use can require intensive care unit admission in case of severe immune-related adverse events (IrAE). Moreover, some of these events, particularly late events, are poorly documented, so a high level of suspicion should be maintained for patients receiving CPI. Intensivists should be aware in general of the known complications and appropriate management of these AE. Nevertheless, a multidisciplinary collaboration remains essential for their diagnosis and management. This review described the most severe complications related to CPI.

**Electronic supplementary material:**

The online version of this article (10.1186/s13613-019-0487-x) contains supplementary material, which is available to authorized users.

## Introduction

**Fig. 1:**
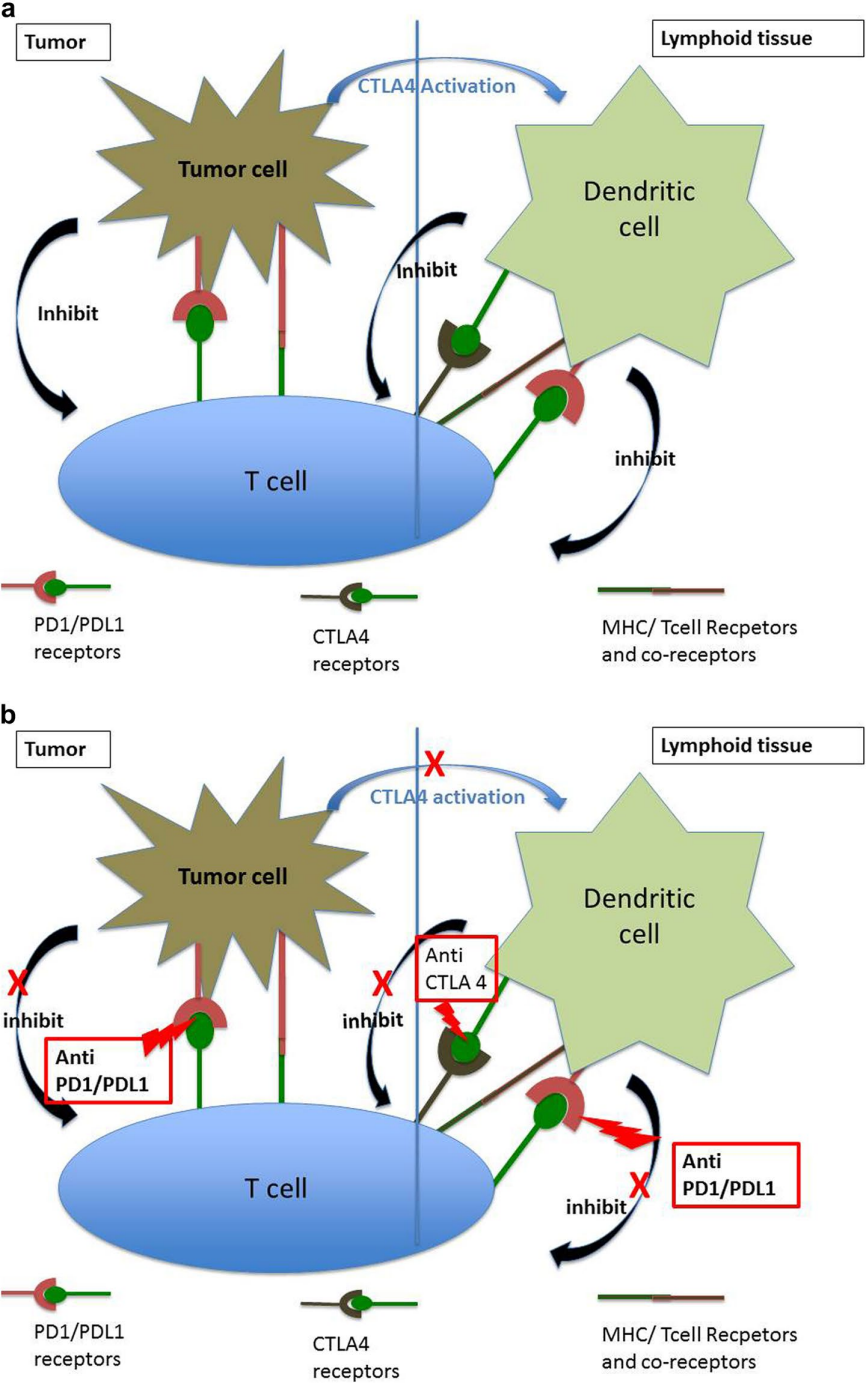
Checkpoint inhibitors: mode of action. **a** Tumor cell inhibition of the immune system: tumor cells decrease T cell activation via two pathways. Within the tumor, connection between tumor cell PDL-1 and T cell receptor PD-1 associated with MHC T cell receptor leads to inhibition of T cell function. Within the lymphoid tissue, tumor cells inhibit dendritic cells via the CTLA-4 pathway. Cancer cells increase CTLA4 expression by dendritic cells, through T reg cell stimulation. Interaction of CTLA4-Receptor on T cell, inhibits T cell function [1]. **b** Mode of action of CTLA-4i or PD-1/PDL-1i. PD-1/PDL-1i blocks the connection between PD-1 and PDL-1 and prevents the inhibition of T cells. T cell cytotoxicity then attacks the tumor cells. CTLA-4i blocks the connection between dendritic cells and T cells related to CTL14. CTLA-4i removes the inhibition related to dendritic cell on T cells

Checkpoint protein inhibitor antibodies (CPI), including cytotoxic T-lymphocyte-associated antigen 4 (CTLA-4) inhibitors (ipilimumab, tremelimumab) and the programmed cell death protein 1 pathway/programmed cell death protein 1 ligand (PD-1/PDL-1) inhibitors (pembrolizumab, nivolumab, durvalumab, atezolizumab), have entered routine practice for the treatment of many cancers. In contrast to classical chemotherapy, CPIs do not target tumor cells; rather they enhance activation of immune cells, particularly T cells (Fig. 1) [1]. They have been associated with better outcomes in a number of solid and hematological malignancies [2]. Moreover, compared with chemotherapy, their tolerance seems to be higher with fewer side effects.

These new molecules are mostly prescribed for melanoma and non-small cell lung cancer (NSCLC), but also for other malignancies such as renal cell carcinoma, bladder carcinoma, squamous cell carcinoma of the head and neck, lymphoma [2]. The list of treatment indications will likely extend as the years go by, even as a first-line therapy. The number of patients treated will increase because of expanded indications and better survival [2–5]. Moreover, the optimal duration of treatment remains unknown.

CPIs are associated with immune-related adverse events (IrAE) that need to be carefully monitored and managed during and after treatment. These drugs can promote infiltration of immune cells into normal tissues, which may lead to immune-mediated disorders. Almost every organ may be affected: skin, bowels, liver, lungs, kidneys, eyes, endocrine tissues, central nervous system [6]. In up to 20% of cases, severe and even life-threatening AE can occur and lead to intensive care unit (ICU) admission [7, 8].

This review focuses on the most severe IrAE that intensivists may encounter. Maintaining a high level of suspicion is a major challenge, as some of the toxicities might be uncovered later, in patients treated for a longer period of time with prolonged survival and new indications for those CPI.

## Methods

We searched Medline and PubMed for reviews and original articles on CPI for treatment of solid tumors in adults published in English between 1 January 2009 and 30 January 2018, using the terms ‘antibody, monoclonal/adverse event’ [Mesh] AND ‘ipilimumab’ [Mesh], ‘tremelimumab’ [Mesh], ‘pembrolizumab’ [Mesh], ‘nivolumab’ [Mesh], ‘durvalumab’ [Mesh], ‘atezolizumab’, [Mesh], and ‘immunotherapy’ [Mesh]. We also searched using individual terms such as ‘CTLA4 inhibitors’, ‘programmed cell death protein 1 pathways’, ‘hepatitis’, ‘pneumonitis’, ‘skin’, ‘hypophysitis’, ‘colitis’, and ‘acute kidney failure’, ‘myocarditis, ‘neurological complication’, ‘encephalopathy’, ‘anemia’. Only severe adverse events eventually associated with ICU admission were considered for this review. Most of the articles were descriptive reports or randomized studies with safety outcomes, over the last 10 years of the search period. We used only original reports when available.

### Statistical analysis

Overall proportion of included study with predefined complication was reported as proportion (95% CI). Publication bias was assessed by visually inspecting the funnel plot, and summary estimates of relative risk and their 95% confidence interval were calculated using both fixed and random-effects model.

Cochran’s *χ*^2^ test and *I*^2^ test for heterogeneity were used to assess inter-study heterogeneity [9]. The *χ*^2^ test assesses whether observed differences among results are compatible with chance alone and the *I*^2^ describes the percentage of the variability in effect estimates that results from heterogeneity rather than from sampling error. An *I*^2^ test for heterogeneity above 0.25 was considered to indicate moderate heterogeneity. Statistically significant heterogeneity was considered present at *χ*^2^ *p* < 0.10 and *I*^2^ > 0.5. All effect sizes with a *p* < 0.05 were considered significant. Tests were two-sided.

All analyses were carried out with software R, version 3.4.4. The ‘meta’, the ‘metasens’ and ‘metaphor’ packages were used to produce forest plots and funnel plots.

## Epidemiology: the magnitude of the problem (Fig. 2)

The severity of AE is defined for each organ by the National Cancer Institute (CTCAE_4.03_2010-06-14_QuickReference_8.5x11.pdf) (Tables 1, 2).

**Fig. 2:**
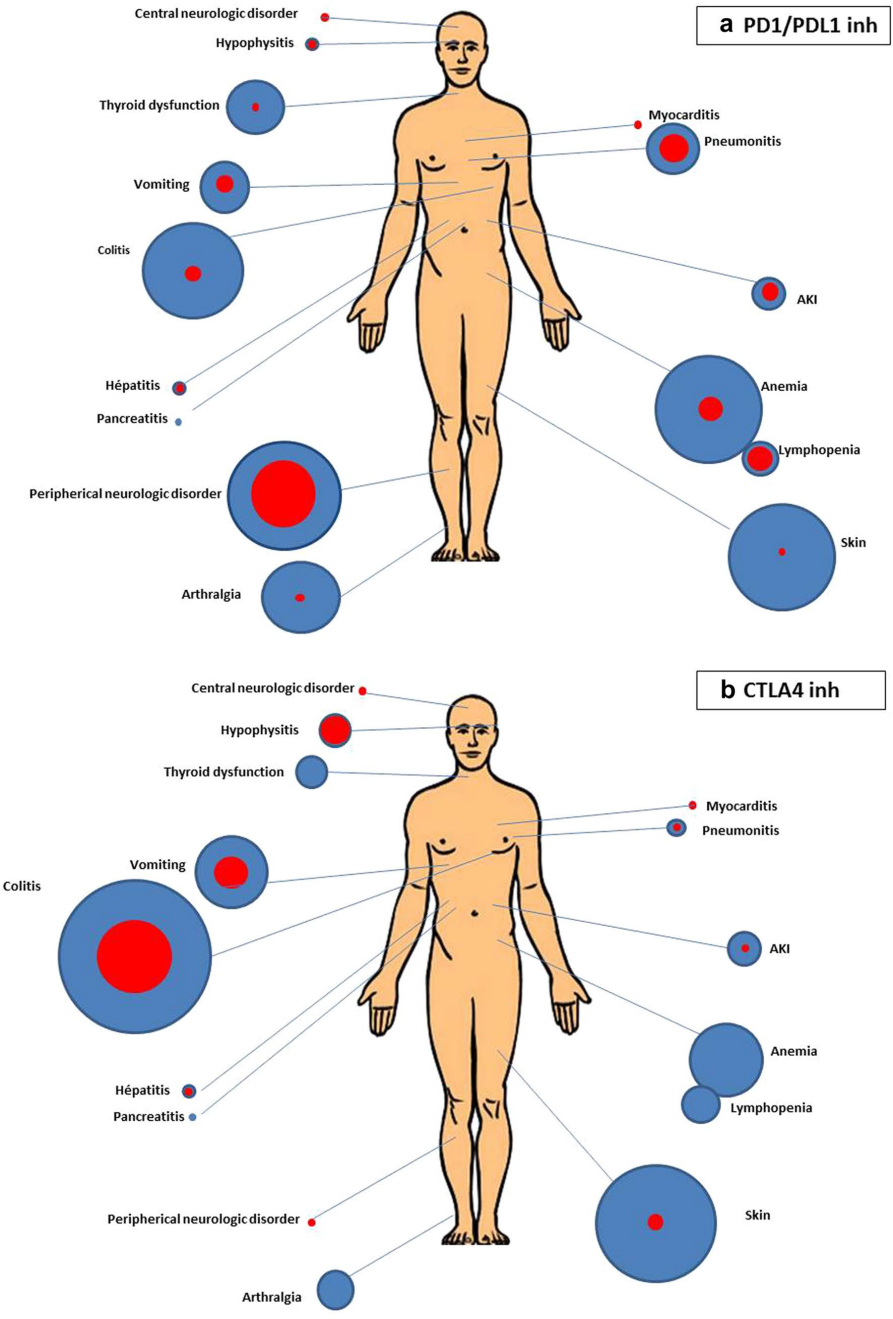
Frequency of immune-related adverse events. **a** Toxicity related to anti-PD-1/PDL-1i; **b** toxicity related to CTLA-4i. The size of the circles reflects the incidence of toxicity: blue, toxicity of any grade; red, grade III/IV toxicity. None of the circles describe toxicity related to CTLA-4i associated with PD-1/PDL-1i inhibitors (the incidence of combined treatments is higher than the toxicity of each inhibitor). Incidence data from [8] and [47]

**Table 1:** Definition of severe immune-related adverse events and diagnostic workup. From [84] and [73] Guidelines from American Society of Clinical Oncology (www.asco.org). CTCAE: common terminology criteria for severe adverse event

Severity of IrAE grade	Outpatient versus inpatient care	Corticosteroids	Others immunosuppressive drugs	immunotherapy
1	Outpatient	Not recommended	Not recommended	Continue
2	Outpatient	Topical steroids or systemic steroids oral (0.5–1 mg/kg/day)	Not recommended	Hold temporarily
3	Inpatient	Systemic steroids oral or i.v. 1–2 mg/kg/day for 3 days then reduce to 1 mg/kg/day	To be considered for patients with unresolved symptoms after 3–5 days of steroid course Organ specialist referral advised	Hold and discuss restarting based on risk/benefit ratio shared with patient
4	Inpatient Consider ICU admission	Systemic steroids i.v. methylprednisolone 2 mg/kg/day for 3 days then reduce to 1 mg/kg/day	To be considered for patients with unresolved symptom after 3–5 days of steroid course specialist consult referral advised	Discontinue permanently

**Table 2:** Management of immune-related adverse events according to severity [85]. Guidelines from American Society of Clinical Oncology (www.asco.org)

Immune-related adverse events	Definitions of severe IrAE	Diagnostic workup before treatment	Steroid and other treatment
*Gastrointestinal* Colitis: disorder with inflammation of colon	Grade III: > 7 stools/day or increase in ostomy output, incontinence, need for hospitalization, limited self-care/ADL Grade IV: life-threatening consequences	Metabolic and hematologic panel TSH *Clostridium difficile*, CMV, parasite CT scan abdomen and pelvis Endoscopy with biopsy	Consider permanently discontinuing CTAL4i. PD/PDL1 agent may be restarted if patient recovered. Consider MP 1–2 mg/kg/day and other immunosuppressive drugs after 3–5 days symptoms
*Lung* Pneumonitis: focal or diffuse inflammation of lung parenchyma (no pathognomonic pattern)	Grade III: severe symptoms, need for hospitalization, more than 50% of parenchyma involved, limited self-care/ADL, need for oxygenation Grade IV: acute respiratory failure with life-threatening consequences	Chest X-ray Thoracic CT Nasal swab, sputum culture, blood and urine culture Bronchoscopy and BAL ± biopsy	Permanently discontinue CPI Empirical antibiotics and 1–2 mg/kg/MP Consider other immunosuppressive drugs after 2 days
*Heart* Myocarditis, pericarditis, arrhythmia, impaired ventricular function, and vasculitis	Grade III: moderate abnormal testing or symptoms occurring with mild activity Grade IV: moderate to severe decompensation, life-threatening consequences	ECG, troponin, BNP Echocardiogram, chest X-ray Cardiac MRI Cardiac catheterization	Permanently discontinue CPI MP 1–2 mg/kg/day Consider other immunosuppressive agent or MP 1 g/day if no improvement
*Neurological* Myasthenia gravis	Grade III–IV: limited self-care, aids warranted, weakness limiting walking, any dysphagia, facial or respiratory weakness or rapidly progressive symptoms	Anti-striated muscle antibody in blood, muscle specific kinase Pulmonary function assessment CPK, CRP ± MRI of spine or brain, EMG	Permanently discontinue CPI Consider MP 2 mg/kg/day MP and plasmapheresis and IVIg 2 g/kg over 5 days
Guillain–Barré syndrome or peripheral neuropathy	Grade III–IV: severe symptoms, limited self-care, aids warranted, weakness limiting walking, any dysphagia, facial or respiratory weakness or rapidly progressive symptoms	Neurological consultation MRI spine Lumbar puncture EMG Pulmonary function testing	Discontinue CPI Consider MP 1–2 mg/kg/day and plasmapheresis
Aseptic meningitis	Grade III–IV: severe symptoms, limited self-care, aids warranted	MRI with pituitary protocol Cortisol and ACTH test Lumbar puncture with measurement of opening pressure	Hold CPI until patient stabilization Consider restarting after risk/benefit analysis. MP 0.5–1 mg/kg
Encephalitis	Grade III–IV: severe symptoms, limited self-care, aids warranted	Neurologic consultation Brain MRI Lumbar puncture EEG CRP, ± , ANCA, TPO, thyroglobulin	Hold CPI until patient stabilization Consider restarting after risk/benefit analysis. Steroid 1–2 mg/kg MP Consider pulse steroids 1 g IV 3–5 days
*Hepatitis*	Grade III: symptomatic liver dysfunction, fibrosis by biopsy, cirrhosis, reactivation of chronic hepatitis, ASAT or ALAT 5–20 N, bilirubin 3–10 N Grade IV: decompensated liver function, ASAT or ALAT > 20 N, bilirubin > 10 N	Viral hepatitis, alcohol history, iron study, thromboembolic event Liver ultrasound (metastasis) ± antinuclear antibody, anti-smooth-muscle antibody, ANCA	Permanently discontinue CPI Steroids 1–2 mg/kg/day MP Consider other immunosuppressive agent after 3 days Avoid using Infliximab
*Endocrine* Hypothyroidism Hyperthyroidism	Grade III–IV: severe symptoms, unable to perform ADL, life-threatening consequences	TSH and T4 dosage	Hold CPI until patient is stabilized Hormone replacement therapy
Adrenal insufficiency	Grade III–IV: severe symptoms, unable to perform ADL, life-threatening consequences	ACTH dosage, cortisol level ± ACTH stimulation test	Hold CPI until patient is stabilized Hormone replacement therapy
Hypophysitis	Grade III–IV: severe symptoms, unable to perform ADL, life-threatening consequences	ACTH dosage, cortisol level +/-ACTH stimulation test TSH and T4 dosage, LH, FSH Brain MRI	Hold CPI until patient has stabilized Hormone replacement therapy
Diabetes mellitus	Grade III–IV: severe symptoms, unable to perform ADL, life-threatening consequences Grade III: blood sugar 13.9–27.8 mmol/l Grade IV: blood sugar > 27.8 mmol/l	Anti-insulin antibody, anti-islet antibody C peptide levels	Hold CPI until glucose control Initiate Insulin therapy
*Kidney* Nephritis	Grade III: creatinine level > 3 × baseline or > 350 µmol/l Grade IV: life-threatening complication, dialysis required	Rule out other causes of AKI Urinary tract infection	Permanently discontinue CPI MP 1–2 mg/kg/day
*Hematologic* Autoimmune hemolytic anemia	Grade III: Hb < 8 g/dl, transfusion indicated Grade IV: life-threatening complication	Drug history, insect bites LDH, haptoglobin, bilirubin, reticulocyte count, autoimmune serology PNH Viral or bacterial infection Protein electrophoresis, cryoglobulin analysis G6PD, methemoglobinemia B12, folate, parvovirus, thyroid ± ADAMTS 13	Permanently discontinue CPI MP 1–2 mg/kg/day Consider other immunosuppressive agent if no improvement.
Immune thrombocytopenia	Grade III: platelet count < 50/mm^3^ Grade IV: platelet count < 25/mm^3^	HIV, hepatitis B or C, *Helicobacter pylori* Reticulocyte count, blood smear ± bone marrow	Hold CPI until improvement Steroid oral 1–2 mg/kg/day Consider IVIg Consider permanently discontinue CPI if no improvement
*Skin* Rash Bullous dermatoses	Grade III: affects quality of life if no response to treatment (rash) Over 30% of body surface (bullous dermatoses) affected, with pain Over 10% of body surface or mucosal involvement (DRESS, pustulosis) Grade IV: > 30% body surface with electrolyte abnormalities (bullous dermatoses) >10% body surface with blood abnormality (liver function)	Whole body examination Assessment for drug, infection Skin biopsy	Permanently discontinue CPI MP 1–2 mg/kg/day Consider other immunosuppressive agent if no improvement

*ADL* activities of daily living, *TSH* thyroid-stimulating hormone, *CMV* cytomegalovirus, *BAL* bronchoalveolar lavage, *MRI* magnetic resonance imaging, *BNP* Brain natriuretic peptide, *CPK* creatine phosphokinase, *CRP* C-reactive protein, *MP* methylprednisolone or equivalent, *EMG* electromyogram, *ANCA* Antineutrophil cytoplasmic *antibodies*, *TPO* thyroid peroxidase, AKI acute kidney failure, *PHN Paroxysmal nocturnal hemoglobinuria*, *ADAMTS 13* a disintegrin and metalloproteinase with a thrombospondin type 1 motif, member 13, *DRESS* drug reaction with eosinophilia and systemic symptoms, *HIV* human immunodeficiency virus

In a meta-analysis of 21 randomized phase II/III immunotherapy trials (including 11,454 patients of whom 6528 received a CPI) conducted between 1996 and 2016, the incidence of fatal IrAE was 0.64%, mostly due to ipilimumab-induced colitis [7].

In patients receiving CPI, grade III–IV (Table 1) colitis occurred in 1.5%, grade III–IV aspartate aminotransferase (AST) elevation in 1.5%, grade III–IV rash in 1.1%, grade III–IV pneumonitis in 1.1%, hypothyroidism was observed in 0.3% of cases. Ipilimumab was associated with a higher risk of grade III–IV colitis than PD-1/PDL-1i [7]. In a recent meta-analysis, PD-1 and PDL-1i seem to be associated with grade III–IV IrAE with similar frequencies [10]. However, the incidence of these IrAE was far lower than the rate of complications from chemotherapy, particularly infections. Grade III–V toxicities were more common with CTLA-4i than with PD-1i (31% vs. 10%) [11]. IrAE leading to death were exceedingly rare for PD-1i (PDL-1i 0.1%, PD-1i 0.3%) and most often secondary to pneumonitis, whereas fatal gastrointestinal (GI) IrAE (diarrhea, colitis, colonic perforation) mostly occurred with CTLA-4i (severe events 31%) [11]. Furthermore, the safety profile of CPI varies among tumor types: melanoma has a higher risk of GI and skin IrAE and lower frequencies of pneumonitis [12, 13]. Moreover, combining two CPIs leads to more frequent severe complications in up to 55% of patients [14–16]. Also, the incidence of rAE and severe IrAE will probably increase in the future, with the increasing number of patients currently treated and the use of combination regimens already tested in several trials [17–19].

The kinetics of IrAE onset remains difficult to describe, but IrAE seem uncommon before 1 months of treatment [6, 13]. Although, in a recent report, severe IrAE can appear early during the treatment course [20] (within 40 days with Ipilimumab and anti-PD1–/PDL1 and 14.5 days with combination treatment), late complications of CPI may occur, sometimes up to 1 year after the start of the PDL1, and clinicians must remain aware of possible complications during follow-up [21]. Moreover, IrAE can occur after the CPI has been discontinued [22].

Toxicities associated with PD-1/PDL-1i agents may be slower to resolve than with ipilimumab, and long-term follow-up is therefore advised [23].

## Immune-related adverse events (Table 2)

**Fig. 3:**
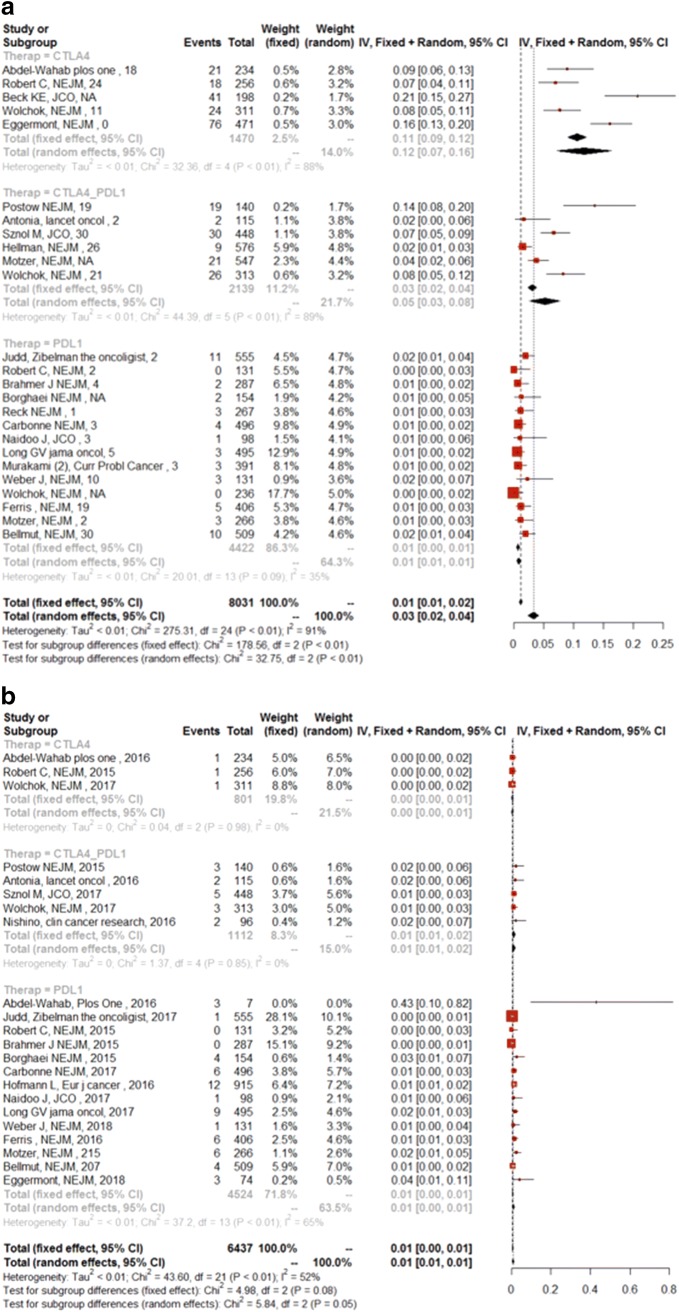
Frequencies of grade III and IV IrAEIrAE in studies. Meta-analysis of randomized control trials including CTLA4i (upper plot), CTLA4i + PD1i/PDL1i (middle plot) or PD1i/PDL1i (lower plot). The forest plots represent the frequencies of IrAEIrAE organ by organ. **a** Severe gastrointestinal irEA; **b** severe lung IrAE. References: [3–5, 13, 16–18, 24, 33, 34, 40, 60, 71, 75, 88–95]

**Fig. 4:**
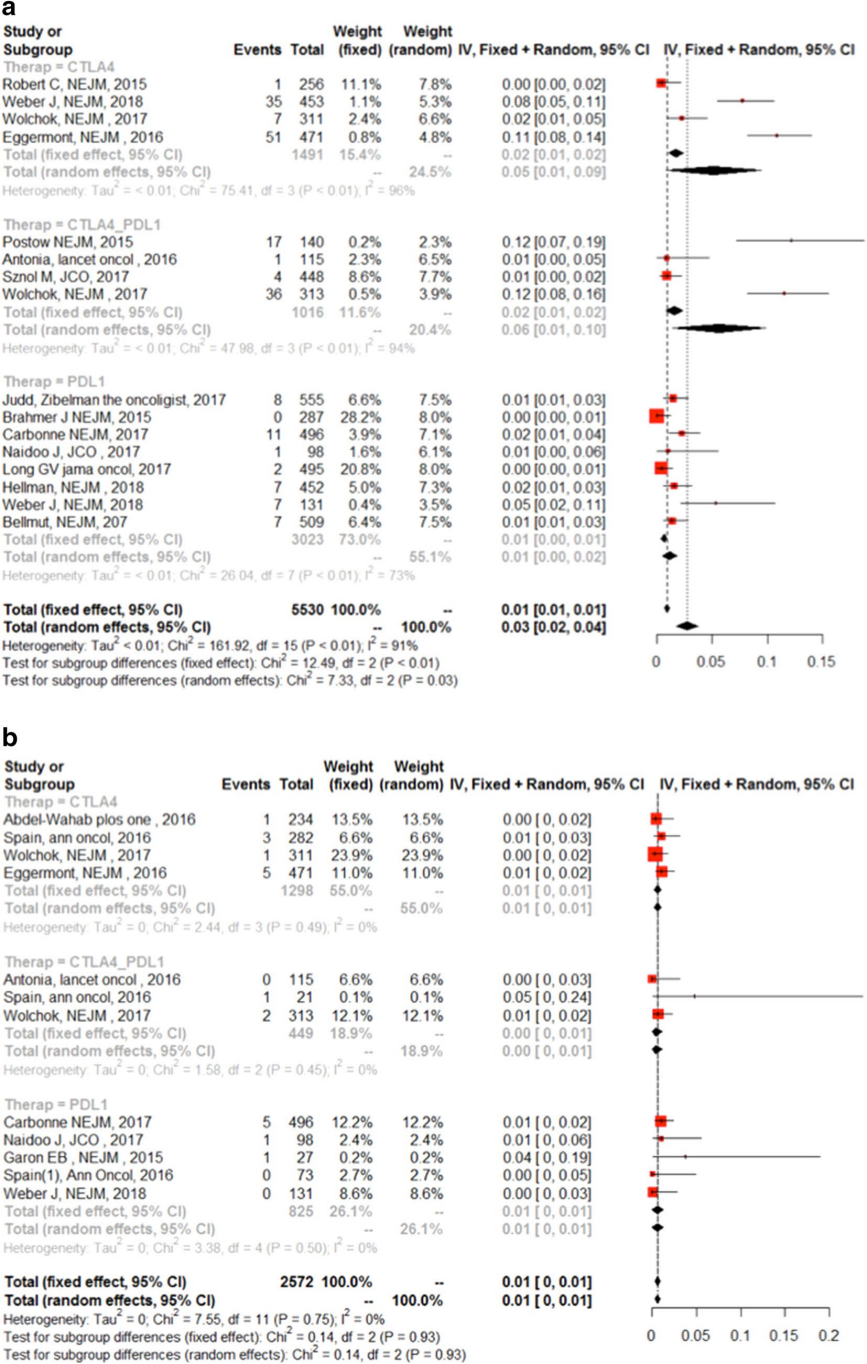
Frequencies of grade III and IV IrAEIrAE in studies. Meta-analysis of randomized control trials including CTLA4i (upper plot), CTLA4i + PD1i/PDL1i (middle plot) or PD1i/PDL1i (lower plot). The forest plots represent the frequencies of IrAEIrAE organ by organ. **a** Severe liver IrAE; **b** severe neurological IrAE. References: [3–5, 13, 16–18, 24, 33, 34, 40, 60, 71, 75, 88–95]

This section describes the most severe IrAE according to the frequency and severity of organ involvement (Figs. 2, 3, 4, Additional file 1: Fig. S1). In some recent studies, high-grade toxicity seems to be associated with high tumoral response rates [24, 25].

### Gastrointestinal disorders

GI disorders are the most frequent IrAE and occur particularly with CTLA-4i. Occurrence of colitis after PD-1i/PDL-1i has been reported only in few patients (< 1%) [23, 26]. At ICU admission, clinicians must distinguish diarrhea alone from colitis. Diarrhea may lead to ICU admission because of dehydration and electrolytes disturbances. Colitis is associated with abdominal pain and inflammation.

Symptoms of GI IrAE have been described in 41/137 patients, largely related to ipilimumab (CTLA4i) [27]. The symptoms can occur within the first few days following the first dose of ipilimumab or weeks after the last dose [20, 26, 27]. On admission, symptoms had been present for 5 days on average (1–64 days), mainly diarrhea (> 90%), abdominal pain (20%), nausea/vomiting (20%), fever (10–12%), anal pain (10%), bleeding (2%), and constipation (2%) [27].

Computed tomography (CT) and/or endoscopy showed evidence of colic inflammation [27]. Endoscopy found histologically confirmed colitis in more than 80% of patients with erythema and ulcerations [27].

Histological examination revealed neutrophilic (46%) and/or lymphocytic (15%) infiltrations, associated in rare cases with abscess and granuloma. These features seem similar to cryptogenic inflammatory bowel diseases [27].

Colitis was in some cases refractory to steroid treatment and led to colonic perforation [27, 28]. In a recent observational study of 21 patients, two patients had refractory colitis lasting for more than 130 days (10 to 12 times the half-life of ipilimumab). Those two patients had previously received radiotherapy. In addition, association of CPI with chemotherapy or other immune therapy may increase the risk of severe colitis [28].

Diarrhea of varying grade occurs frequently in patients treated with CTLA-4i. However, alternative diagnoses should be evaluated at ICU admission. First, an infectious etiology should be excluded, particularly *Clostridium difficile* (Table 2). The incidence of *C. difficile* in CPI-treated patients remains unknown, with only a small number of cases described [29, 30]. The diagnostic workup must include at least stool culture and screening for *C. difficile* and cytomegalovirus (PCR and/or colon biopsy). CT and endoscopy should be performed if possible to distinguish colitis from other possible diagnoses (bowel cancer, other inflammatory bowel disease, etc.).

Although GI adverse events related to PD-1i are rare, severe colitis has been described after long-term PD-1i treatment [26].

### Lung disorders, pneumonitis/acute respiratory distress syndrome

Although pneumonitis remains rare (4% in NSCLC and 3% in melanoma), it can lead to severe acute respiratory distress syndrome (ARDS) (0.8–1% grade 3 or higher toxicity in the studies included) [31–33]. Rare cases of severe pneumonitis have been described in phase I trials with PD-1i and PDL-1i [34, 35]. CTLA-4i are rarely associated with pneumonitis although some cases series have shown non-severe [36] or severe pneumonitis [37]. Pneumonitis is more frequent during NSCLC treatment than melanoma treatment, particularly when other lung process is present (tobacco use, chronic obstructive pulmonary disease, etc.) or during combined treatment [33]. Pneumonitis should be distinguished from cancer relapse or infection [38]. One case report described a “flare pneumonitis” after tapering corticosteroids without new treatment with PD-1i [39]. More interestingly, in a descriptive study of 43 cases of pneumonitis related to PD-1/PDL-1i, more than half of the patients described other immune toxicity as well [40]. Common symptoms included dyspnea (53%), cough (35%), fever (12%), and chest pain (7%). ARDS occurred in rare cases [40].

**Fig. 5:**
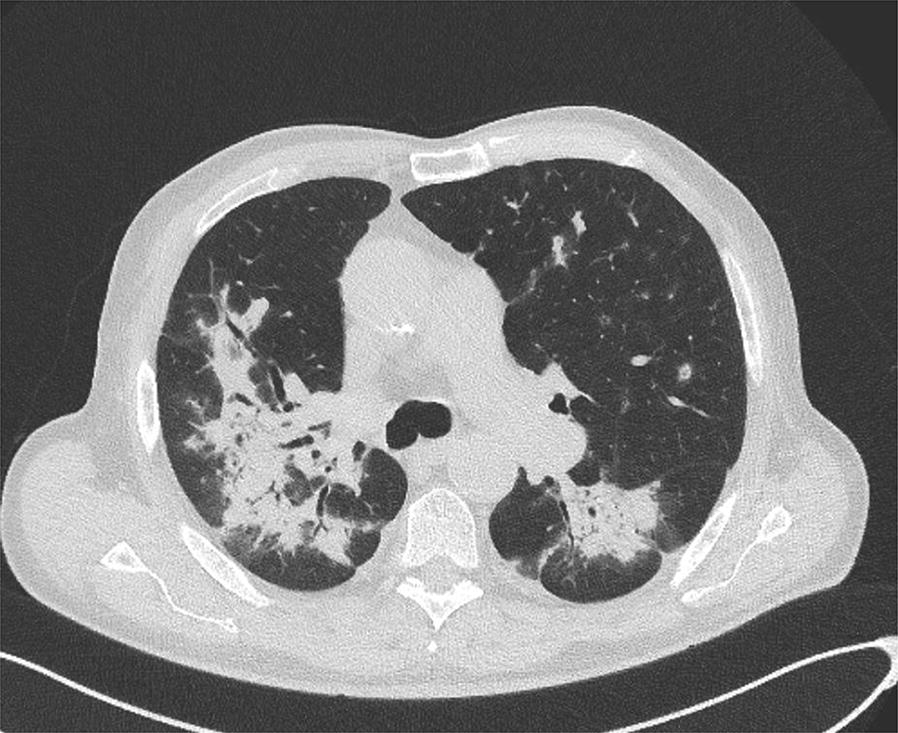
Example of thoracic CT in immune-related pneumonitis. The patient had been treated for NSCLC with pembrolizumab for 2 months. He developed acute respiratory failure. CT showed the typical organizing pneumonia pattern

PD-1i-related pneumonitis was described in 20 of 170 patients treated with PD-1i. Among them, five patients had severe pneumonitis occurring within 2.6 months after the beginning of treatment. Cough was the most frequent symptom, followed by dyspnea and fever. The most frequent CT findings were ground-glass opacities in all patients, reticular opacities (19/20 patients) and airspace consolidation (12/20 patients), with a common organizing pneumonia pattern in 13 (65%) patients (Fig. 5). Abnormal findings occurred in the lower lobes with a peripheral distribution [33, 39, 41]. Another CT pattern encountered was non-specific interstitial pneumonia. Unfortunately, none of the studies described the findings of bronchoalveolar lavage (BAL). Other causes of acute respiratory failure (infection including, *Pneumocystis jirovecii* pneumonia, relapsing cancer etc.) must be excluded. BAL should be performed in those cases. In one study, lung biopsy was performed in 11 patients. The histological findings were cellular interstitial pneumonitis, common organizing pneumonia, or diffuse alveolar damage. In three patients, no lesion was found [40].

Pneumonitis related to ipilimumab is rare but has been reported as sarcoidosis/granulomatosis-like, rarely associated with ARDS [36, 37].

Management of patients with suspected grade III–IV pneumonitis should include clinical examination to search for other associated immune toxicities, leading to higher probability of IrAE pneumonia, and CT should be performed to define the lesions. BAL and potentially lung biopsy should be considered (Table 2).

### Myocarditis and cardiac insufficiency

Myocardial complications remain rare, far below the rate of toxicities related to radiotherapy and chemotherapy. However, cases reports described grade III/IV IrAE, ranging from cardiomyopathy to acute myocarditis and cardiac arrest [42, 43]. This rare complication remains one of the most severe consequences and occurs more frequently with the combination of CTLA-4i and PD-1i or PDL-1i [44].

It may occur at the initiation of therapy or after several weeks of treatment. Cardiovascular risk factors (e.g. hypertension and tobacco use) were not always present in cases reports of cardiac toxicity [45, 46]. Interestingly, in a recent study of eight cases, five patients had already at least one other IrAE when the cardiac side effect occurred [47].

The best management of CPI-related myocarditis remains unknown. Wang et al. proposed an algorithm to detect and treat myocarditis, including pre-treatment troponin and EKG [45]. Other causes of myocardial dysfunctions should be ruled out (pulmonary embolism, ischemic myocardial dysfunction) (Table 2). Treatment may require extracorporeal membrane oxygenation, infliximab, or polyvalent intravenous immunoglobulins [44, 47, 48].

Some cases of pericardial effusion, sometimes with tamponade, have also been reported [49–51]. A few rare cases of pericarditis occurred that were treated with steroids. When histological examination was performed, T cell infiltrates were found with cardiomyocytes fibrosis, in some cases, [46].

### Neurologic disorders: encephalopathy, Guillain–Barré syndrome, myasthenia, myelitis

Because of the severity of symptoms, neurological toxicity remains one of the most important IrAE, mostly associated with CTLA-4i [52]. Although these complications are common, the proportion of grade III/IV cases remains limited. Neurological complications may appear within 4 months after initiation of treatment, but clinicians should maintain a high level of awareness even when these drugs were recently introduced. Furthermore, they are usually prescribed for a long duration which could lead to delayed toxicity. Some cases reports describe permanent disability after neurological toxicity. Central neurotoxicity can take several forms, from headache after ipilimumab induction to chronic encephalopathy or aseptic meningitis [53, 54]. Stroke and posterior reversible encephalopathy syndrome after ipilimumab may occur and could lead to ICU admission [52]. Seizures remain rare [54–56], peripheral neurotoxicity occurs with Guillain–Barré syndrome or neuromyopathy, after CTLA-4i or anti-PDL-1i treatment [52, 53]. Myasthenic syndromes may occur mostly with PD1i treatment [57, 58], early after treatment initiation [59] (Table 2). Other etiologies, particularly metastases, should be ruled out with MRI and or lumbar puncture.

In an observational study of 352 patients with melanoma, 10 patients were found to have severe neurological (central and peripheral) complications (including six patients with high-grade complications). Eight of those patients showed a sustained response to steroid therapy and were alive after 8 to 35 months [53]. The high survival rate after neurological CPI justifies ICU admission, but other etiologies should be promptly ruled out as well.

### Endocrine-related adverse events

Endocrine-related AE are irreversible IrAE and lead to continuous substitutive treatment. Failure to diagnose these IrAE can lead to life-threatening complications particularly hypophysitis and adrenal insufficiency. Higher incidences of endocrine-related AE were found with combination therapy and when high dose therapy was used [60].

#### Thyroid dysfunction

Thyroid dysfunction, isolated or associated with hypophysitis, occurred in up to 10% of cases and was severe (grade III/IV) in only 1–2% of patients [43, 61]. Thyroid dysfunction (hypothyroidism or hyperthyroidism) could be primary or secondary in origin. Associated hypophysitis should be considered particularly with CTLA-4i [61].

Although thyrotoxicosis has been described in rare cases, primary hypothyroidism is more frequent than hyperthyroidism and mostly related to PD-1i or PDL-1i treatment. Hashimoto’s disease has been described in rare cases [6]. Other associated endocrinopathies should also be considered [60].

#### Hypophysitis

First described with CTLA-4i, hypophysitis can also occasionally occur with PD-1i and anti-PDL-1i treatment. Intensivists must be aware of this complication, which can be life-threatening particularly when acute adrenal insufficiency is the first symptom. Hyponatremia and dehydration may lead to ICU admission. Adrenal insufficiency is much more frequent with immunotherapy than with conventional treatment [53].

Hypophysitis was investigated in 211 patients treated with CTLA4i for melanoma and developed early in the course of the treatment. Hypophysitis occurred in 19 (9%) patients within 4 months of treatment and was symptomatic in 83% of these cases. Associated hypothyroidism occurred in 11 (58%) patients, while brain magnetic resonance imaging revealed abnormal findings in only 12 (63%) patients [62]. Hypophysitis seems to be related to hypersensitivity of hypophysis cells carrying CTLA-4 receptors [63].

#### Other endocrine adverse events

Primary adrenal insufficiency remained rare (< 1%) [62].

Severe diabetes mellitus with ketosis was described in 0.4% of cases with PDL-1i. Diabetes was related to pancreatic disorder or to autoimmune insulin-dependent diabetes. (Table 2) [64, 65]. Ketoacidosis may require ICU admission [64].

### Liver disorders

Liver dysfunction, mostly related to autoimmune-like hepatitis, has been described with CTLA-4i treatment but very rarely with PD-1i or PDL-1i treatment [66, 67]. In a case series of 11 patients receiving one to four doses of ipilimumab, the authors described acute panhepatitis with CD8 + T-lymphocyte perivenular infiltrate and endothelialitis. Some of the patients had pre-existing risk factors for chronic liver disease with nonalcoholic steatohepatitis or steatosis-associated characteristics. CT showed hepatomegaly and periportal edema. Viral hepatitis (VH), tumor progression, and autoimmune hepatitis should be excluded by biological diagnostic testing including testing for hepatitis A, B, C or E, antinuclear antibodies, smooth-muscle antibodies, and anti-mitochondrial antibodies (Table 2). No case of fulminant hepatitis has yet been described [66, 68] with CTLA-4i treatment. CMV-related hepatitis coupled with colitis has been described [69].

One case of fulminant hepatitis has been described within 34 weeks after PD-1i was started, but no liver biopsy was performed [70]. The patient improved with steroids.

The rate of PD-1i related hepatitis grade III was 0.5% during melanoma treatment and has not been described during lung cancer treatment [4, 71].

### Pancreatic disorders

In a recent study of 496 patients treated for melanoma, pancreatitis disorders occurred in 9 (0.02%) patients including seven patients with grade III/IV pancreatitis, within 6 to 20 weeks after treatment initiation [6]. In earlier studies, elevated lipase was reported in less than 1% of patients [4, 71].

Some authors recently demonstrated that high lipase level was not associated with pancreatic disease in most cases and should not be automatically associated with treatment cessation [72].

### Skin

Skin involvement is frequent: 50% of patients experiment rash or pruritus with CTLA-4i and 22% with PD-1i [73]. However, grade III/IV IrAE are very rare, reported in 0 to 4% of patients after ipilimumab treatment [13] and even more rarely with PD-1/PDL-1i treatment [74, 75]. Stevens–Johnson syndrome is one of the most severe complications (Table 2).

### Kidney disorders

According to clinical trials, acute kidney injury (AKI) is relatively uncommon with anti-cancer immune CPI compared with other types of IrAE [76]. However, both circulating anti-double-stranded DNA antibodies and glomerular IgG and C3 deposits have been reported in mice treated with CTLA-4i [77].

During ipilimumab monotherapy, elevated creatinine was reported in 1.4% (any grade) and 0.2% (grade III or IV) of the patients. Similarly, during PD-1i monotherapy, elevated creatinine was reported in 1.7% (any grade) and 0.8% (grade III or IV) of the patients, respectively. However, during combination therapy, the incidence of AKI was higher in clinical trials, resulting in 1.7% of grade III or IV creatinine elevation.

The most accurate data were reported in the series by Cortazar et al. and Shirali et al. [78, 79]. They reported the clinical and histological features of 13 patients with CPI-related AKI (various cancers, mainly melanoma; various CPIs) who underwent kidney biopsy. The most prevalent pathologic lesion was acute tubulo-interstitial nephritis in 12 patients, including three with granulomatous features, and one case of thrombotic microangiopathy (TMA) (Table 2).

The renal prognosis remains good after discontinuing CPI and in most cases prescription of steroids (Table 2). However, the persistence of kidney failure after 3 weeks, higher age, and greater degree of interstitial fibrosis have been associated with poor prognosis [80]. In some reports, interstitial fibrosis may occur as soon as 10 to 14 days after initiation of treatment.

### Hematological syndromes

Rare cases of hemolytic anemia leading to ICU admission have been described with nivolumab and ipilimumab, IgG or C3 mediated [81]. They may respond to corticosteroids, but rituximab may be required in some cases. Resumption of PD-1i/PDL-1i after resolution of anemia was not always associated with a recurrence of anemia [82, 83] (Table 2).

## Management

Due to the potential reversibility with treatment, CPI-related severe toxicity should lead to ICU admission at least for a time-limited trial, in case of organ failure or for patients at risk of organ failure. Such a trial may include mechanical ventilation, vasopressors, renal replacement therapy, and even extracorporeal membrane oxygenation in selected patients.

The recommendations for managing IrAE arise from general clinical consensus, because no prospective trials have been conducted to specifically test whether one management strategy is superior to another. Early recognition and treatment of IrAE are believed to be important in mitigating their severity. For severe grade III–IV, IrAE drug should be discontinued immediately [84].

**Fig. 6:**
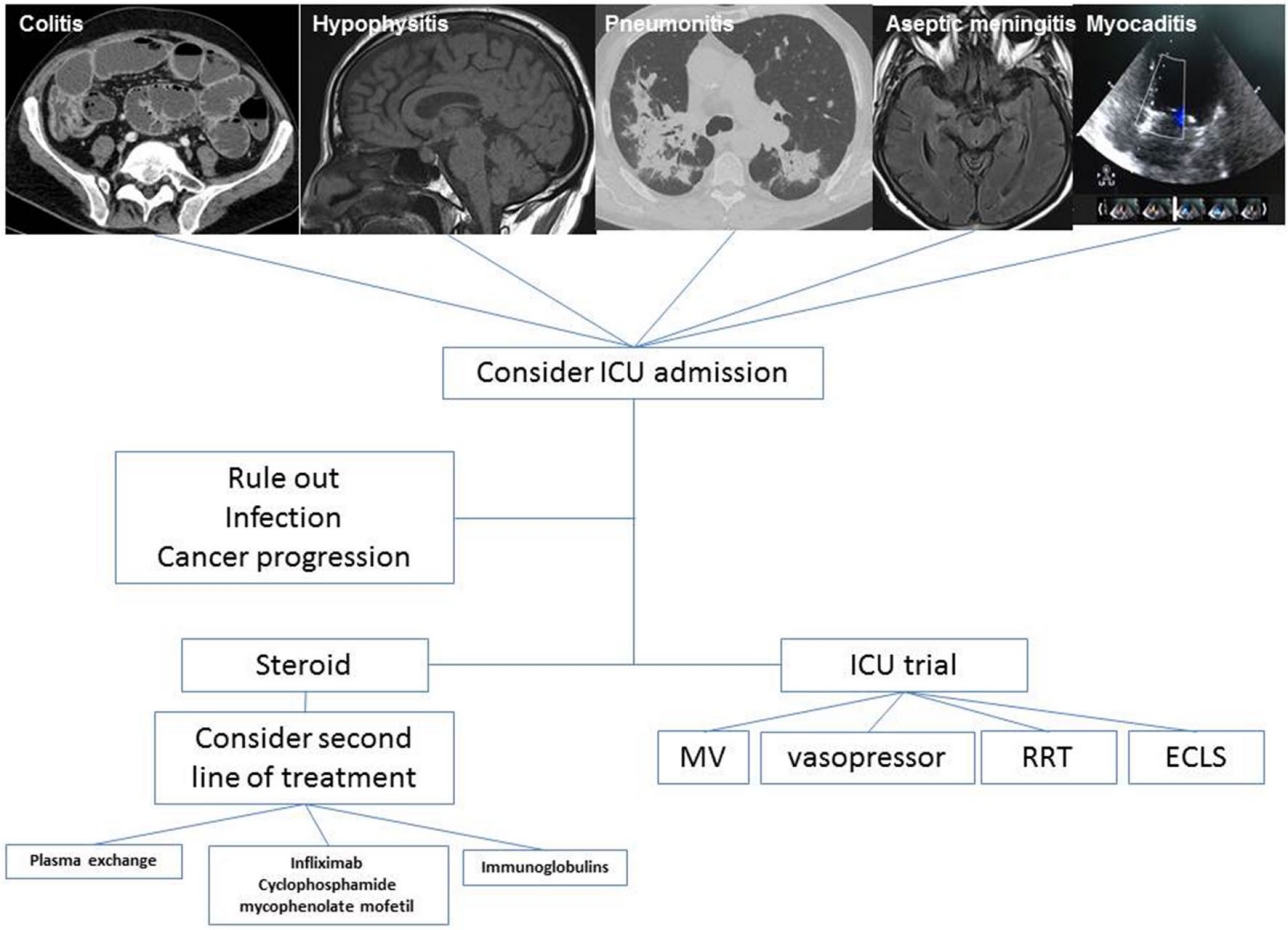
Management of the most frequent IrAEs in the ICU. *ICU* intensive care unit, *MV* mechanical ventilation, *RRT* renal replacement therapy, *ECLS* extracorporeal life support

From a practical standpoint, the management of such patients requires a close collaboration between specialists (e.g. nephrologist, hepatologist, infectious diseases specialist), oncologists, and intensivists (Fig. 6).

Although steroid treatment should be initiated as soon as possible, some other etiologies like infections or cancer progression must be ruled out. Table 2 summarizes the diagnostic work up before treatment. Most of the differential diagnoses can be ruled out quickly and work up should not delay the initiation of steroid treatment in case of severe IrAE. Systemic corticosteroids (oral or IV methylprednisolone) must be initiated at a dose of 1–2 mg/kg/day for 3 days and then reduced to 1 mg/kg/day. Corticosteroids regimen should then follow a gradual tapering over a period of at least 1 month [85]. Whenever IrAEs worsen or do not improve sufficiently after 3–5 days despite the use of adequate steroids dosage, additional immunosuppressive drugs should be considered. Although none of these treatments has been evaluated, they may include:

- Antitumor necrosis factor alpha (anti-TNF) [86] in case of colitis or pneumonitis, but not hepatitis because of the risk of hepatotoxicity [8].
- Mycophenolate mofetil (500–1000 mg twice a day) for hepatitis, cardiotoxicity, or pneumonitis [8, 45].
- Antithymocyte immunoglobulins for hepatitis, cardiotoxicity, or severe neurotoxicity [8, 45].

Hypothyroidism should be managed with thyroid hormone replacement and hyperthyroidism with standard anti-thyroid pharmacotherapy and beta-blockers in symptomatic cases [8].

Long-term treatment with corticosteroids and sometimes anti-TNF drugs may be complicated by severe opportunistic infections such as fungal infection, tuberculosis, or CMV. Therefore, it is recommended to give antibiotic prophylaxis with oral trimethoprim/sulfamethoxazole (400 mg/125 mg 3 times a week) together with steroids and to test patients for tuberculosis before adding any additional immunosuppressive drug (e.g. TNF alpha inhibitors) to corticosteroids [85.].

As the pathophysiological mechanism of IrAE involves excessive activation of the immune system, leading to toxic effects potentially targeting any organ and mimicking autoimmune diseases in their clinical presentation, it may also be difficult to differentiate between the side effects of CPI and the development of autoimmune paraneoplastic syndromes. This was emphasized in a recent report of patients without previous autoimmune manifestations who developed autoimmune encephalitis during immunotherapy [55, 87].

Interestingly, the same CPI may be reintroduced after IrAEIrAE resolution in most cases of grade III IrAEIrAE (Table 2). For grade IV IrAEIrAE, resumption of CPI may be more questionable. However, restarting CPI must be considered after a close collaboration between oncologist, specialist and intensivist, while weighing the individual risk/benefit ratio, and should be shared with the patient (Table 2).

Interestingly, some studies described higher cancer response rate for patients who also experienced high-grade IrAEIrAE [13]. These results need to be confirmed.

## Conclusion

Severe immune-related complications of checkpoint protein inhibitor antibodies complications remain rare, but the number of patients treated will continue to rise. Although adverse events may occur less frequently after immunotherapy than after chemotherapy, intensivists should be aware of the side effects of this new type of medication that may require ICU admission. Moreover, some immune-related complications remain unknown and will reveal themselves with the increasing use of those new therapeutic agents.

## Additional file

**Additional file 1: Fig. S1.** Frequencies of grade III and IV IrAE in studies: meta-analysis of randomized control trials including CTLA4i (*upper plot*), CTLA4i + PD1i/PDL1i (*middle plot*), or PD1i/PDL1i (*lower plot*). The forest plots represent the frequencies of IrAE organ by organ. **a** Severe dermatologic IrAE; **b** severe endocrine IrAE; **c** severe myocardiac IrAE; **d** severe hematological IrAE; **e** severe renal IrAE; **f** severe peripheral neuropathic IrAE. References: [3–5, 13, 16–18, 24, 33, 34, 40, 60, 71, 75, 88–95].

## Abbreviations

CPI: checkpoint protein inhibitors
PD-1: programmed cell death protein 1
PD-1i: programmed cell death protein 1 inhibitors
PDL-1: programmed cell death protein 1 ligand
PDL-1i: programmed cell death protein 1 ligand inhibitors
CTLA-4: cytotoxic T-lymphocyte antigen 4
CTLA-4i: cytotoxic T-lymphocyte antigen 4 inhibitors
IrAE: immune-related adverse events
