# Supplementary figures and images for: Severe toxicity from checkpoint protein inhibitors: What intensive care physicians need to know?
Source: Ann Intensive Care. 2019 Feb 1;9:25. doi: 10.1186/s13613-019-0487-x (PMC6358632; doi:10.1186/s13613-019-0487-x)

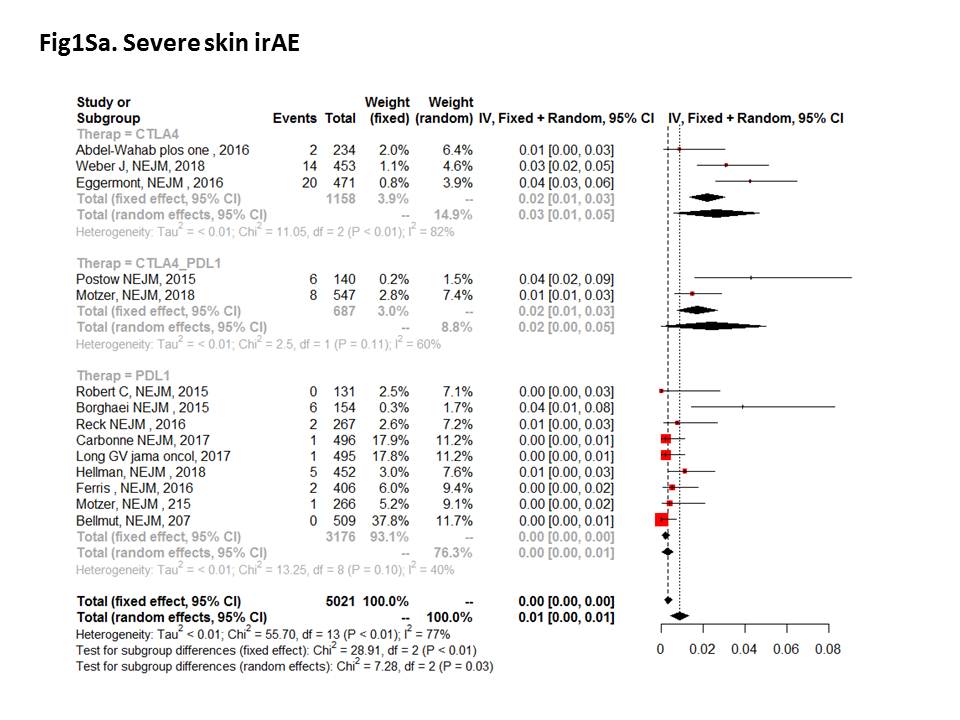

Supplement: Supplementary file 1 — Additional file 1: Fig. S1. Frequencies of grade III and IV IrAE in studies: meta-analysis of randomized control trials including CTLA4i (upper plot), CTLA4i + PD1i/PDL1i (middle plot), or PD1i/PDL1i (lower plot). The forest plots represent the frequencies of IrAE organ by organ. a Severe dermatologic IrAE; b severe endocrine IrAE; c severe myocardiac IrAE; d severe hematological IrAE; e severe renal IrAE; f severe peripheral neuropathic IrAE. References: [3–5, 13, 16–18, 24, 33, 34, 40, 60, 71, 75, 88–95]. [file 13613_2019_487_MOESM1_ESM.zip › 13613_2019_487_MOESM1_ESM/Fig1Sa.JPG]

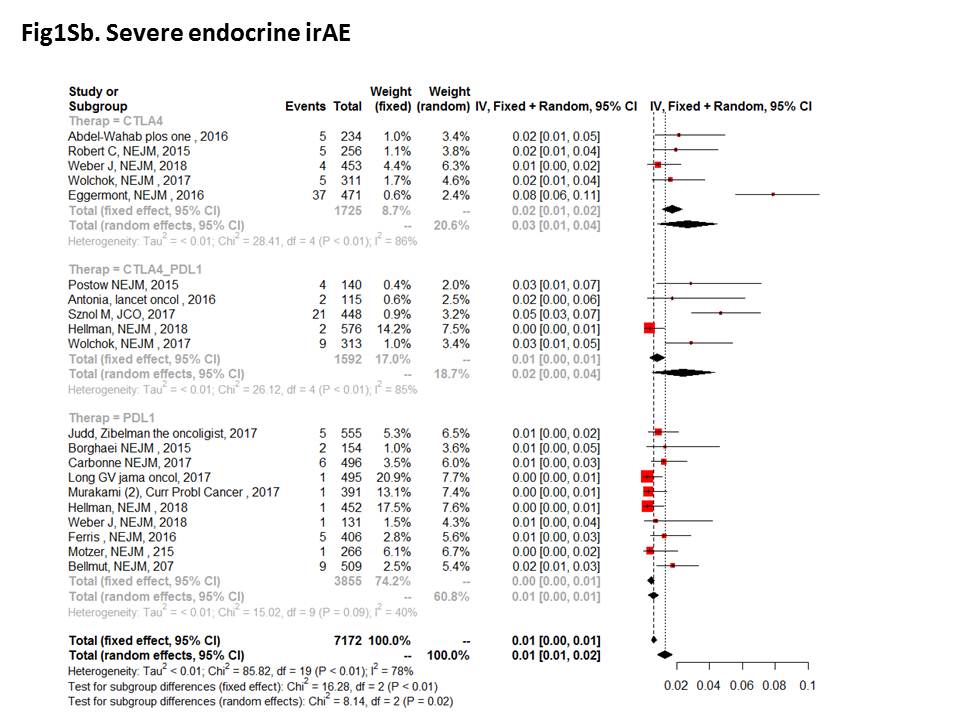

Supplement: Supplementary file 1 — Additional file 1: Fig. S1. Frequencies of grade III and IV IrAE in studies: meta-analysis of randomized control trials including CTLA4i (upper plot), CTLA4i + PD1i/PDL1i (middle plot), or PD1i/PDL1i (lower plot). The forest plots represent the frequencies of IrAE organ by organ. a Severe dermatologic IrAE; b severe endocrine IrAE; c severe myocardiac IrAE; d severe hematological IrAE; e severe renal IrAE; f severe peripheral neuropathic IrAE. References: [3–5, 13, 16–18, 24, 33, 34, 40, 60, 71, 75, 88–95]. [file 13613_2019_487_MOESM1_ESM.zip › 13613_2019_487_MOESM1_ESM/Fig1Sb.JPG]

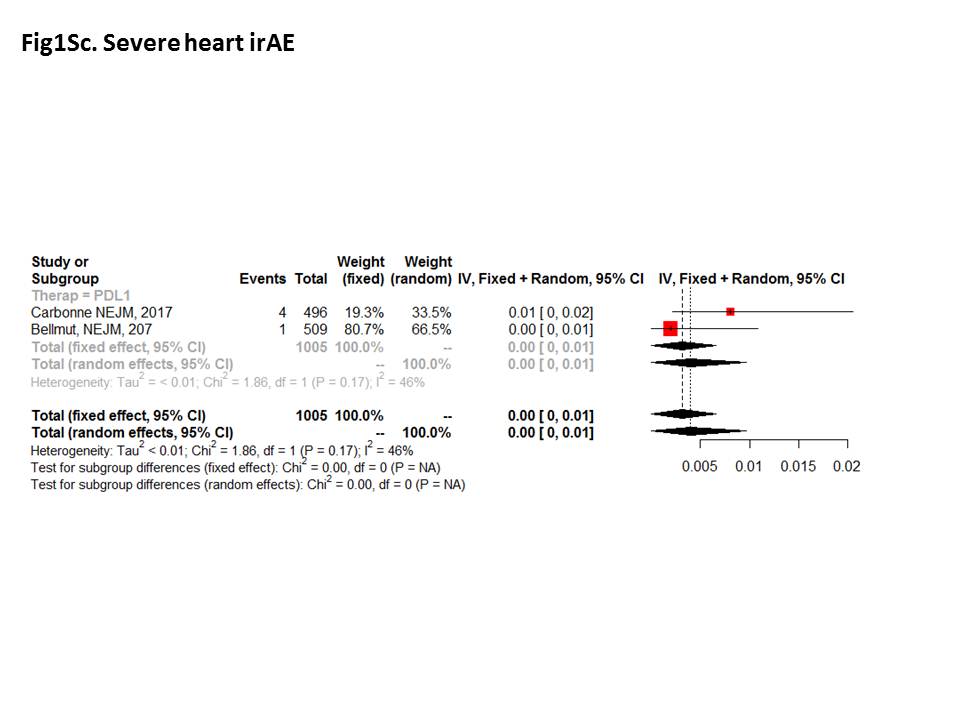

Supplement: Supplementary file 1 — Additional file 1: Fig. S1. Frequencies of grade III and IV IrAE in studies: meta-analysis of randomized control trials including CTLA4i (upper plot), CTLA4i + PD1i/PDL1i (middle plot), or PD1i/PDL1i (lower plot). The forest plots represent the frequencies of IrAE organ by organ. a Severe dermatologic IrAE; b severe endocrine IrAE; c severe myocardiac IrAE; d severe hematological IrAE; e severe renal IrAE; f severe peripheral neuropathic IrAE. References: [3–5, 13, 16–18, 24, 33, 34, 40, 60, 71, 75, 88–95]. [file 13613_2019_487_MOESM1_ESM.zip › 13613_2019_487_MOESM1_ESM/Fig1Sc.JPG]

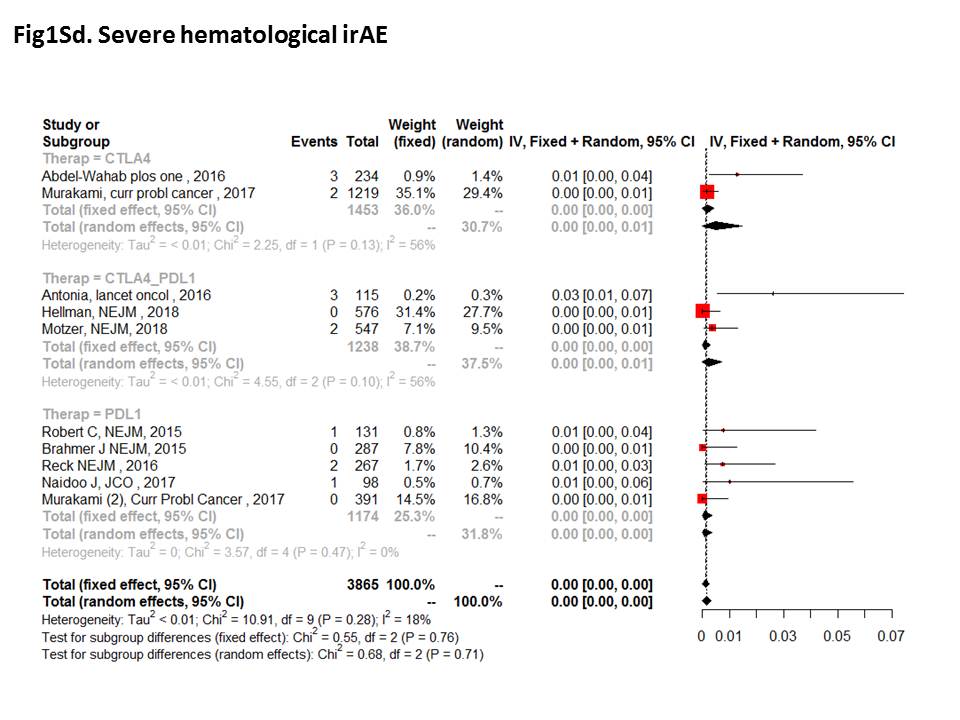

Supplement: Supplementary file 1 — Additional file 1: Fig. S1. Frequencies of grade III and IV IrAE in studies: meta-analysis of randomized control trials including CTLA4i (upper plot), CTLA4i + PD1i/PDL1i (middle plot), or PD1i/PDL1i (lower plot). The forest plots represent the frequencies of IrAE organ by organ. a Severe dermatologic IrAE; b severe endocrine IrAE; c severe myocardiac IrAE; d severe hematological IrAE; e severe renal IrAE; f severe peripheral neuropathic IrAE. References: [3–5, 13, 16–18, 24, 33, 34, 40, 60, 71, 75, 88–95]. [file 13613_2019_487_MOESM1_ESM.zip › 13613_2019_487_MOESM1_ESM/Fig1Sd.JPG]

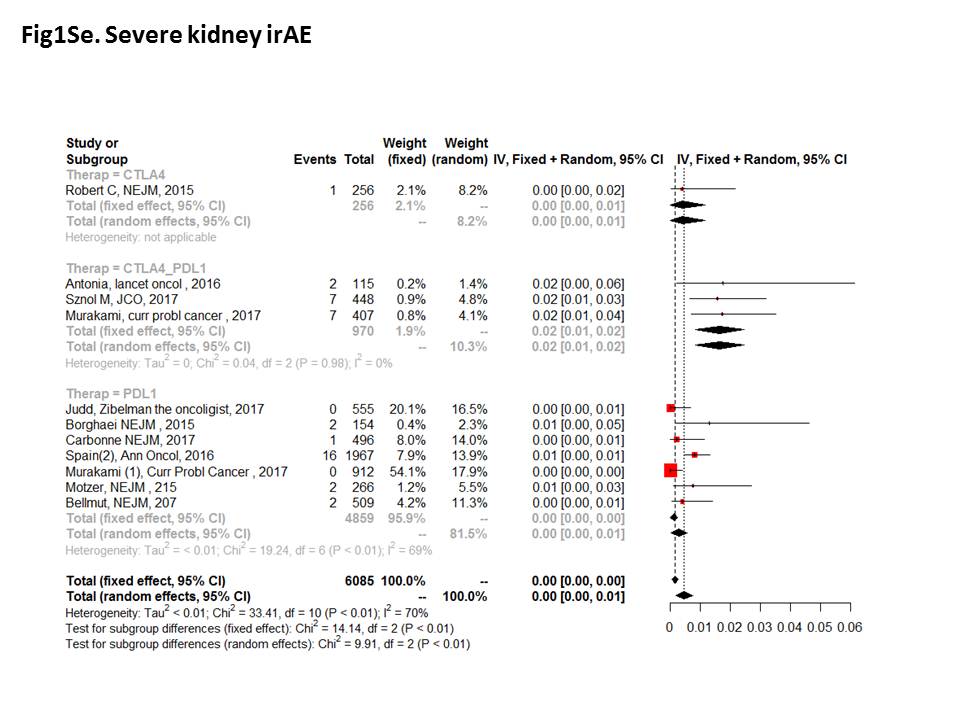

Supplement: Supplementary file 1 — Additional file 1: Fig. S1. Frequencies of grade III and IV IrAE in studies: meta-analysis of randomized control trials including CTLA4i (upper plot), CTLA4i + PD1i/PDL1i (middle plot), or PD1i/PDL1i (lower plot). The forest plots represent the frequencies of IrAE organ by organ. a Severe dermatologic IrAE; b severe endocrine IrAE; c severe myocardiac IrAE; d severe hematological IrAE; e severe renal IrAE; f severe peripheral neuropathic IrAE. References: [3–5, 13, 16–18, 24, 33, 34, 40, 60, 71, 75, 88–95]. [file 13613_2019_487_MOESM1_ESM.zip › 13613_2019_487_MOESM1_ESM/Fig1Se.JPG]

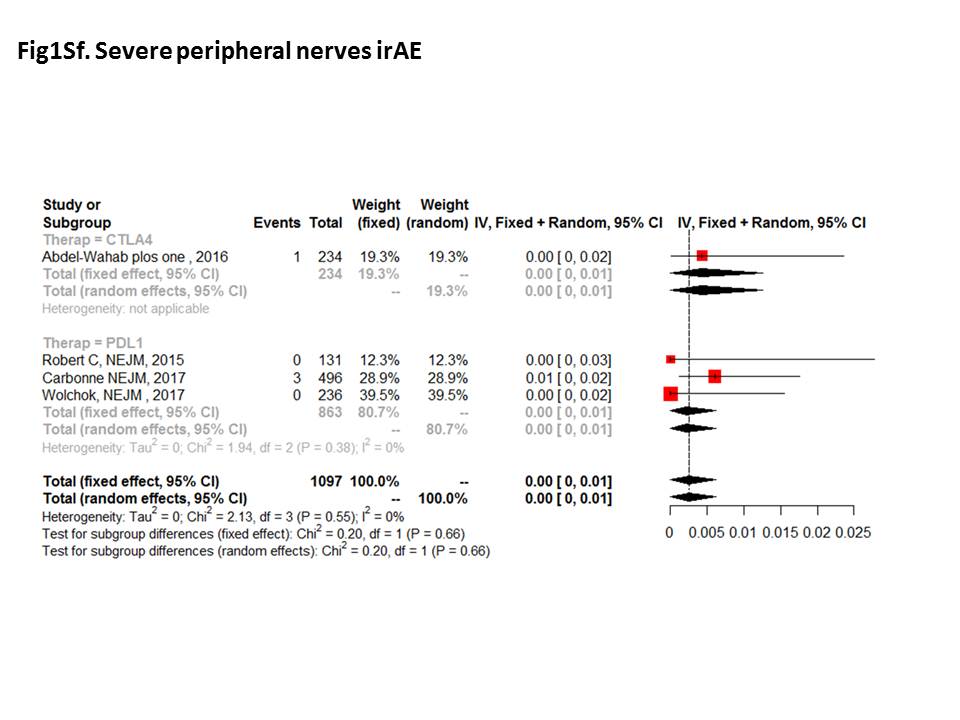

Supplement: Supplementary file 1 — Additional file 1: Fig. S1. Frequencies of grade III and IV IrAE in studies: meta-analysis of randomized control trials including CTLA4i (upper plot), CTLA4i + PD1i/PDL1i (middle plot), or PD1i/PDL1i (lower plot). The forest plots represent the frequencies of IrAE organ by organ. a Severe dermatologic IrAE; b severe endocrine IrAE; c severe myocardiac IrAE; d severe hematological IrAE; e severe renal IrAE; f severe peripheral neuropathic IrAE. References: [3–5, 13, 16–18, 24, 33, 34, 40, 60, 71, 75, 88–95]. [file 13613_2019_487_MOESM1_ESM.zip › 13613_2019_487_MOESM1_ESM/Fig1Sf.JPG]
